# Attrition in the Canadian Healthy Infant Longitudinal Development (CHILD) study

**DOI:** 10.1186/1710-1492-10-S1-A16

**Published:** 2014-03-03

**Authors:** JC Venevongsa, R Chooniedass, AL Kozyrskyj, CD Ramsey, AB Becker

**Affiliations:** 1 Department of Pediatrics and Child Health, University of Manitoba, Winnipeg, MB, Canada; 2 Manitoba Institute of Child Heath, Winnipeg, MB, Canada; 3 Department of Pediatrics, University of Alberta, Edmonton, AB, Canada; 4 Community Health Sciences, University of Manitoba, Winnipeg, MB, Canada; 5 Department of Medicine, University of Manitoba, Winnipeg, MB, Canada

## Background

Studies have shown that retention of study subjects is a major challenge in research. Longitudinal studies provide a wealth of information over time, but participants need to continue until the study is complete. Study participants have the right to withdraw.

## Objective

1. To determine the attrition rate across study sites in the CHILD study.

2. To document the principle reasons for attrition.

3. To identify maternal factors associated with attrition

## Methods

CHILD is a study assessing the environmental impact on children’s health. The study includes 4 recruitment sites across Canada (Vancouver, Edmonton, Manitoba, and Toronto). Women enrolled while pregnant will be followed, along with their child for 5 years. Home assessments were done at 3 months, clinical assessments at 1, 3, and 5 years of age, and questionnaires are administered every 6 months. If a study participant withdraws, staff completes a one page questionnaire to determine reasons for withdrawal. The checklist incudes: no reason given, father not interested, family lacks time, family concern regarding privacy, expense to family, inconvenient to travel, complicated family situation, testing difficult, enrolled in another study, too many questionnaires, separation, personal health issue and others. Maternal factors included: history of asthma or food allergy, marital status, socioeconomic status (post secondary education, income), ethnic/ cultural group, being born in Canada, age, and stress measured by a Perceived Stress Scale.

## Results

CHILD recruited 3628 participants at 4 sites (Vancouver 816, Edmonton 840, Manitoba 1107, and Toronto 865). Of the 316 participants withdrawn from the study, 149 were not eligible due to birth issues and 167 withdrew). Attrition rates for those 167 who declined further study were 4.5% (37/816) in Vancouver, 6.2% (52/840) in Edmonton, 2.3% (25/1107) in Manitoba, and 6.1% (53/865) in Toronto. Of the 167, further information is available only for 100. 81% (81/100) withdrew due to a lack of time, 17% (17/100) withdrew due to family issues, and 16% (16/100) due to other reasons, such as moving away, religions reason, etc. We compared 2698 participants with available data with 167 withdrawn active participants. Perceived stress was high in 99% withdrawn vs. 69% of active participants (p=0.02). Other important factors for withdrawing include single mother status (12% vs. 6%, p=0.03), maternal history of asthma (13% vs. 23%, p=0.03), and maternal food allergy (11% vs. 22%, p=0.009).

## Conclusion

Lack of time and family issues were the main reasons for withdrawal. There was greater attrition among mothers who were single parents, had greater stress, and who did not have asthma and allergy. There were no significant differences for education, income, cultural group, being born in Canada or maternal age.

## Relevance

It is important to retain families for longitudinal studies. Researchers must be aware of the main reasons and factors associated with attrition before designing longitudinal studies, and implement retention effective strategies.

